# Antibody Cross-Reactivity between Porcine Cytomegalovirus (PCMV) and Human Herpesvirus-6 (HHV-6)

**DOI:** 10.3390/v9110317

**Published:** 2017-10-28

**Authors:** Uwe Fiebig, Angela Holzer, Daniel Ivanusic, Elena Plotzki, Hartmut Hengel, Frank Neipel, Joachim Denner

**Affiliations:** 1 Robert Koch Institute, Nordufer 20, 13353 Berlin, Germany; FiebigU@rki.de (U.F.); IvanusicD@rki.de (D.I.); elena.plotzki@gmx.de (E.P.); 2 Institute of Virology, University Erlangen, Schlossgarten 4, 91054 Erlangen, Germany; Angela.Holzer@viro.med.uni-erlangen.de (A.H.); Frank.Neipel@viro.med.uni-erlangen.de (F.N.); 3 Institute of Virology, Medical Center, Faculty of Medicine, University of Freiburg, Hermann-Herder-Strasse 11, 79104 Freiburg, Germany; Hartmut.Hengel@uniklinik-freiburg.de

**Keywords:** porcine cytomegalovirus, human cytomegalovirus, xenotransplantation, virus transmission, human herpesvirus-6

## Abstract

Porcine cytomegalovirus (PCMV) infection is widely prevalent among pigs, and PCMV is one of the viruses which may be transmitted during xenotransplantation using pig cells, tissues, or organs. While human cytomegalovirus (HCMV) is a major risk factor for allotransplantation, it is still unclear whether PCMV is able to infect human cells or pose a risk for xenotransplantation. Previously, it was shown that transmission of PCMV after pig kidney to non-human primate transplantations resulted in a significantly reduced survival time of the transplanted organ. To detect PCMV, PCR-based and immunological methods were used. Screening of pigs by Western blot analyses using recombinant viral proteins revealed up to 100% of the tested animals to be infected. When the same method was applied to screen human sera for PCMV-reactive antibodies, positive Western blot results were obtained in butchers and workers in the meat industry as well as in normal blood donors. To exclude an infection of humans with PCMV, the sera were further investigated. PCMV is closely related to human herpesvirus-6 (HHV-6) and human herpesvirus-7 (HHV-7), and a sequence alignment of glycoprotein B suggests that the antibodies may cross-react with identical epitope sequences. HCMV is not related with PCMV, and no correlation between antibody reactivity against PCMV and HCMV was detected. These data indicate that antibodies against PCMV found in humans are cross-reactive antibodies against HHV-6.

## 1. Introduction

Herpesviruses are double-stranded DNA viruses with a diameter of 150–200 nm, causing diseases in animals as well as in humans. In humans, nine herpesviruses were found, herpes simplex viruses 1 and 2 (HSV-1 and HSV-2, also known as HHV-1 and HHV-2), varicella-zoster virus (VZV, HHV-3), Epstein-Barr virus (EBV or HHV-4), human cytomegalovirus (HCMV or HHV-5), two variants of the human herpesvirus 6 (HHV-6A and HHV-6B), human herpesvirus 7 (HHV-7), and Kaposi's sarcoma-associated herpesvirus (KSHV, also known as HHV-8) [1]. Herpesviruses were also found in many other species, including pigs [2]. Suid herpesvirus-1 (SuHV-1) corresponds to the pseudorabies virus, SuHV-2 to the porcine cytomegalovirus (PCMV), and SuHV-2, -3, and -4 to the porcine lymphotropic herpesviruses (PLHV)-1, -2, and -3. SuH1 belongs to the subfamily alphaherpesvirinae, and PLHVs belong to the subfamily gammaherpesvirinae, genus *Macavirus* [2]. PCMV was recently defined as a betaherpesvirus, genus *Roseolovirus* [3]. This implies that PCMV is more closely related to the human roseoloviruses HHV-6 and HHV-7 compared with the namesake human cytomegalovirus (HCMV, or HHV-5) [3].

In the context of virus safety of xenotransplantation using pig cells, tissues, or organs as replacement for human transplants, PCMV may be transmitted to the recipient (for review see [4]). Xenotransplantation is under development due to the increasing shortage of human transplants, and this new technology has made significant progress in the last years [5,6]. Whether PCMV represents a risk factor for human xenotransplant recipients is still unclear. HCMV, a betaherpesvirus, genus *Cytomegalovirus*, is well known as a major risk factor for human allotransplantation and an important cause of morbidity and mortality in immunocompromised individuals [7].

It still remains controversial whether PCMV is able to infect and replicate in human cells, as has been demonstrated for other non-human cytomegaloviruses, such as mouse CMV [8]. In one report, PCMV was shown to infect primary human fibroblasts, to induce cytopathogenic effects in human cells, and to transcribe PCMV genes resulting in the synthesis of PCMV proteins [9], whereas in another report, human kidney 293 and Raji B cell lines did not support PCMV replication [10]. 

Evidence for a putative risk posed by PCMV in xenotransplantation was obtained when kidneys from PCMV-infected and uninfected pigs were transplanted into cynomolgus monkeys and baboons. In both cases the survival time of the transplant from the infected pigs was drastically reduced compared with the transplant from uninfected pig donors [11,12]. Although there was no evidence of a direct infection of non-human primate cells with PCMV, the dysfunction and transplant failure were interpreted to result from PCMV infection or replication [11,12]. Transmission of PCMV was also observed after pig heart transplantation, which was associated with injury of the transplant and an increased incidence of consumptive coagulopathy [13]. Early weaning excluded PCMV, resulted in a prolonged survival of the transplant and prevented consumptive coagulopathy [13].

To evaluate the possibility that PCMV may occasionally infect humans, human sera, mainly from individuals having close contact with pigs, were analyzed for PCMV-reactive antibodies as an indirect evidence of infection. To study the antibody response a newly developed Western blot assay comprising four recombinant protein sequences, two corresponding to the N-terminal and C-terminal part of the glycoproteinB (gB) of PCMV [14] and two newly produced tegument proteins, were applied as antigens. In addition, an analysis of the sequences of PCMV and other herpesviruses was performed.

## 2. Materials and Methods

### 2.1. Recombinant Antigens and Western Blot Analysis for the Detection of PCMV-Specific Antibodies

Two recombinant proteins corresponding to the N-terminus (nucleotides, nt, 539–929) and C-terminus (nt 2771–3118, Acc. No: AF268039) of the sequence of the glycoprotein gB of PCMV were used as antigens [14]. These domains are highly conserved among the 46 sequences of PCMV analyzed. Both sequences were expressed as 10× His-tagged recombinant fusion proteins in *E. coli* BL21cells (New England Biolabs, Frankfurt am Main, Germany) and purified by affinity chromatography using HisTrap columns (GE Healthcare, Buckinghamshire, UK). The tegument proteins U54A and U54B of PCMV [3] were expressed and purified as follows: The U54A sequence is located at position 70307–72304 (protein ID: AGT99246.1, GenBank No. KF017583) and the sequence of U54B is located at position 72345–73541 (protein ID: AGT99247.1, GenBank No. KF017583). The sequences were codon-optimized by the JAVA codon adaptation tool (JCAT) algorithm for *E. coli* expression [15] and synthesized by ATGbiosynthetics (Merzhausen, Germany). The synthetic gene sequences were cloned into the expression vector pet16b (Novagen, Madison, WI, USA) using the restriction enzymes *Nde*I and *Xho*I (New England Biolabs). The cloned sequences were confirmed by Sanger sequencing. Both sequences were expressed as recombinant His-tagged fusion proteins in *E. coli* BL21cells (New England Biolabs). The transformed *E. coli* cultures were diluted from an overnight culture to an optical density at 600 nm wavelength (OD_600_) of 0.1 in 2 L 2YT-Medium (1.0% yeast extraxt, 1.6% tryptone, pH 7.0). The *E. coli* cultures were then grown at 37 °C until they reached an OD_600_ of 0.7, followed by induction with 1 M isopropyl β-d-1-thiogalactopyranoside (IPTG). After 3 h of induction, cells were pelleted at 8000 rpm for 15 min and stored at −20 °C until purification. *E. coli* cell pellets were resuspended in buffer phosphate-buffered saline (PBS), 1 mg/mL lysozyme, Sigma-Aldrich, St. Louis, MO, USA, and 50 µL DNase, Thermo Fisher, Waltham, MA, USA), sonicated three times for 20 s, and incubated on ice for 20 min. The cell debris was removed by centrifugation (10,000 rpm, 10 min) and pellets were extracted with lysis buffer (6 M guanidinium chloride, 500 mM NaCl, 20 mM disodium phosphate, pH 7.5) for 1 h under shaking at room temperature. Solubilized proteins were separated from the remaining insoluble material by centrifugation (25,000 rpm, 20 min), diluted to 100 mL with lysis buffer, and loaded on HisTrap 5 mL excel columns (GE Healthcare, Buckinghamshire, UK). The columns were equilibrated with lysis buffer and loaded with solubilized proteins. After washing with lysis buffer and a second wash buffer (8 M urea, 500 mM NaCl, 15 mM imidazole, 20 mM disodium phosphate, pH 7.5) the proteins were eluted using a 10-column volume gradient with elution buffer (8 M urea, 500 mM NaCl, 500 mM imidazole, 20 mM disodium phosphate, pH 7.5). 

The Western blot analysis was performed as described previously [14,16], using 500 ng/lane His-tagged gB protein. The proteins were dissolved in sample buffer (50 mM Tris-HCl, 12% glycerol, 4% sodium dodecyl sulfate (SDS), 5% β-mercaptoethanol, 0.01% bromophenol blue) and denaturated for 5 min at 95 °C prior to electrophoresis, and then analyzed using 10% or 14% polyacrylamide gel and as a molecular weight marker of the PageRuler pre-stained protein ladder (Thermo Fisher). Proteins were transferred for 50 min to nitrocellulose membranes by electroblotting (15 V) and stained with Ponceau red, cut into strips, and blocked over night at 4 °C with 5% blotting grade dry milk (Carl Roth, Karlsruhe, Germany) in PBS with 0.05% Tween 20 (blocking buffer). Strips were incubated with sera diluted 1:300 in blocking buffer for 2 h at room temperature. Polyclonal goat anti-pig immunoglobulin G (IgG)-alkaline phosphatase (AP) (Abcam, Cambridge, UK) was diluted 1:1000 in blocking buffer. For the detection of the gB and tegument proteins, a 1:1000 dilution of the Penta-His antibody (Qiagen, Hilden, Germany) as the primary antibody and 1:1000 polyclonal rabbit anti-mouse IgG-horseradish peroxidase (HRP) or IgG-AP (Dako, Hamburg, Germany) were used as well. Staining was performed with 3,3′-diaminobenzidine (DAB) (Thermo Fisher) or with 5-bromo-4-chloro-3-indolyl-phosphate nitro blue tetrazolium (NBT/BCIP) (Promega, Madison, WI, USA). All Western blot analyses were repeated two to five times.

### 2.2. Sera

Sera from 11 Göttingen minipigs, which are the result of cross-breeding the Minnesota minipig, the Vietnamese potbelly pig, and the German Landrace pig (Ellegaard Göttingen Minipigs A/S, Dalmose, Denmark, http://www.minipigs.dk/), from 12 Aachen minipigs, which are based on cross-breeding minipigs derived from different private minipig farms located mainly in Eastern Germany [17] and 12 animals for slaughter from an abattoir near Berlin, Germany (slaughterhouse pigs), were collected and stored at −80 °C. Sera from butchers and individuals working in the meat production industry as well as sera from healthy blood donors tested previously for antibodies against porcine endogenous retroviruses (PERV) [18] were used. Cytotect (Biotest, Dreiech, Germany) was used, which is a licensed anti-HCMV polyclonal immunoglobulin preparation from human plasma from HCMV-positive individuals. In addition, 10 human sera samples were analyzed, which had been shown to be positive or negative for HCMV antibodies using the LIAISON CMV IgG II assay (DiaSorin, Saluggia, Italy). Finally, two human sera samples negative for HHV-6 and four human sera samples positive for HHV-6 antibodies, as shown by an immunofluorescence assay, were analyzed. The use of human blood was approved by the ethics commission at the Medical Faculty of the Humboldt University Berlin. Written informed consent was provided by study participants.

### 2.3. Nucleotide Sequence Alignment

In order to confirm the immunological cross-reactivity between PCMV (accession number AF268039), HHV-6A (AAA43846), HHV-6B (ARM61233), and HHV-7 (YP073779) at the sequence level, an alignment of the sequences of the glycoproteins of human herpes viruses and PCMV was performed using the software DNASTAR Lasergene 10, Clustal W method [19].

### 2.4. Assay for the Detection of Antibodies against HCMV

Screening for HCMV-specific antibodies was performed at the Laboratory “Labor Berlin” of the Charite and the Vivantes hospitals, Berlin, using the Abbott Architect CMV IgG chemiluminescent microparticle immunoassay (CMIA). At the University of Freiburg, Faculty of Medicine, HCMV-reactive antibodies were estimated using the LIAISON CMV IgG II assay (DiaSorin).

### 2.5. Immunofluorescence Assays for the Detection of Antibodies against HHV-6 and HHV-7

Antibodies against HHV-6 were screened for binding by an indirect immunofluorescence assay using HHV-6 strain U1102-infected and uninfected HSB-2 cells, as previously described [20]. Briefly, infected cells were harvested 4 to 5 days after infection, washed in PBS, air-dried on slides, and fixed for 10 min in ice-cold acetone. Fixed cells were incubated with either human or porcine sera at dilutions of 1:16 and 1:64 in PBS for 30 min at 37 °C. After removal of the diluted sera, the slides were washed twice for 5 min in PBS at room temperature with gentle agitation. This was followed by incubation with fluorescein isothiocyanate (FITC)-conjugated goat anti-human or anti-pig IgG for 30 min at 37 °C. After washing in PBS as above, the cells were briefly counterstained with a 1:20,000 dilution of Evans Blue and mounted with 50% glycerol in PBS. Slides were examined using a Zeiss fluorescence microscope (Carl Zeiss, Oberkochen, Germany) at 250× magnification. All assays were repeated at least two times.

### 2.6. Real-Time PCR for the Detection of PCMV

A duplex real-time PCR was performed as described previously [14], using primers specific for PCMV (forward (fw) ACTTCGTCGCAGCTCATCTGA, 45206–45226, KF017583.1, reverse (rev) GTTCTGGGATTCCGAGGTTG, 45268–45249 and probe 6FAM-CAGGGCGGCGGTCGAGCTC-TAMRA, 45246–45229) [21] and porcine glycerinaldehyd-3-phosphat-dehydrogenase (GAPDH) (fw ACATGGCCTCCAAGGAGTAAGA, 1040–1062 NM_001206359.1, rev GATCGAGTTGGGGCTGTGACT, 1188–1168, probe HEX-CCACCAACCCCAGCAAGAG-BHQ1, 1114–1132) [22]. The TaqMan Universal PCR 2× Mastermix (Life Technologies, Carlsbad, CA, USA) and 100 ng of DNA isolated from blood using the DNeasy blood and tissue kit (Qiagen) were used.

## 3. Results

### 3.1. Detection of Antibodies against PCMV in Pig and Human Sera

**Figure 1:**
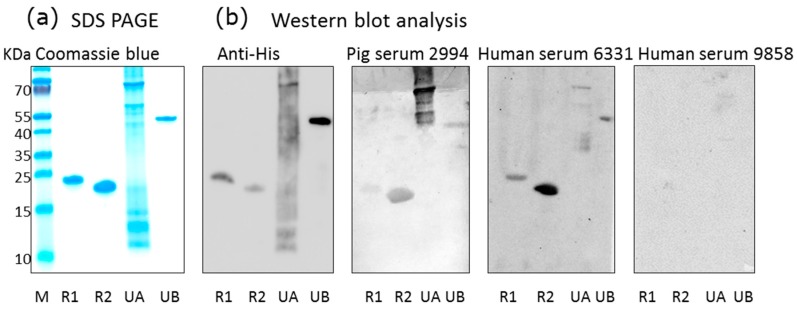
Western blot analysis of human and pig sera using porcine cytomegalovirus (PCMV) proteins as antigens. (**a**) SDS-PAGE and Coomassie blue staining of purified recombinant PCMV-derived proteins: R1, N-terminal part of gB, R2, C-terminal part of gB, UA, tegument protein U54A, UB, tegument protein U54B, M, marker proteins; (**b**) Western blot analysis using antibodies against the His-tag, pig serum 2994, human sera 6331 and 9858. Antibodies were used at the following dilutions: anti-His antibodies 1:1000, pig serum 2994 1:300, human sera 6331 and 9858 were used 1:300, anti-mouse serum 1:1000, anti-human serum 1:1000, and anti-pig serum 1:1000.

Using a recently developed Western blot assay based on two recombinant protein sequences corresponding to the N-terminus and C-terminus of the glycoprotein B of PCMV [14], as well as two newly produced PCMV tegument proteins, sera from different pig breeds were screened for PCMV-specific antibodies (Figure 1). With exception of the U54A tegument protein, all recombinant proteins were stable, as shown by sodium dodecyl sulfate polyacrylamide gel electrophoresis (SDS-PAGE) (Figure 1a), and all were detected by an antiserum against the His-tag (Figure 1b). When pig sera were screened, all four recombinant PCMV-derived proteins were detected by some pig sera, among them were pigs confirmed to be PCMV-infected by PCR. Negative pig sera did not react with the PCMV proteins. Surprisingly, also some human sera reacted with the PCMV proteins, for example serum from individual 6331, whereas others (individual 9858) did not react (Figure 1b).

**Figure 2:**
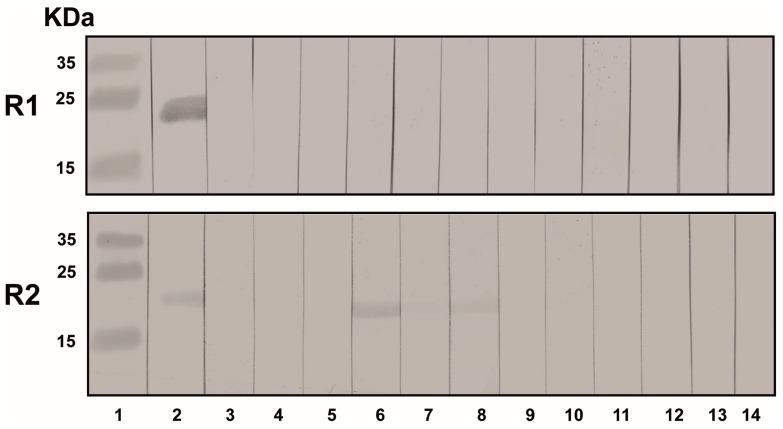
Western blot analysis of sera from humans working as butchers or in the meat industry using the R1 and R2 recombinant protein of glycoprotein B (gB) of PCMV. Anti-His serum was used as a positive control (lane 2). Anti-His serum was used at a dilution of 1:1000, human sera at 1:300, and anti-human serum at 1:1000. Lane 1, marker proteins, lane 2, antibodies against the His tag, lane 4 to 14, different human sera samples.

Altogether, 35 pig sera samples from three different pig breeds and 48 sera samples from humans working as butchers, having close contact with pigs, or working in the meat industry as well as healthy blood donors were tested. Twenty-six pigs and four humans were found to be positive (Figure 2, Table 1). Some of the human sera samples analyzed here were the same as those that previously tested negative for PERV transmission [18].

### 3.2. Sequence Comparison of PCMV and Other Herpesviruses

**Figure 3:**
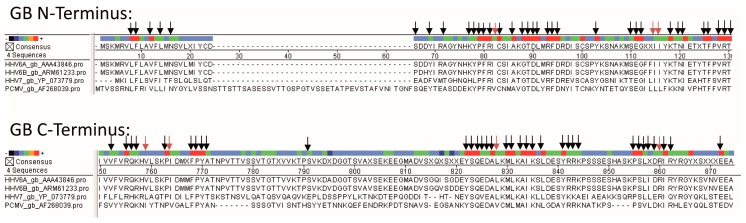
Multiple alignment of protein sequences of gB of PCMV, HHV-6A, HHV-6B, and HHV-7. gB N-terminus, N-terminal part of gB of PCMV corresponding to R1, gB C-terminus, C-terminal part of gB of PCMV, corresponding to R2. The accession numbers are indicated. Dots represent missing amino acids and deletions. Arrows indicate related amino acids between PCMV and HHV-6A (black arrows, identical amino acids; red arrows, conserved amino acids, R=K, L=I=V, F=Y).

The presence of antibodies in human sera suggests that either the human individuals are infected with PCMV or the antibodies are cross-reacting with a human herpesvirus. The seroprevalance of human herpesviruses is relatively broad worldwide. Specifically, HSV-1 50–90%, HSV-2 4–60%, VZV 5–80%, CMV 52–99%, HHV-6 39–100%, HHV-7 96%, EBV 66–85%, and KSHV 1–60% of the population are seropositive [23,24]. In individuals infected with the human immunodeficiency virus HIV-1, more and multiple herpesviruses have been described. EBV, HHV-8, CMV, and HSV-1 were detected in 90%, 57%, 31%, and 16% of samples compared with 48%, 24%, 2%, and 2% of samples from controls [24]. Phylogenetic studies showed that PCMV belongs to the genus *Roseolovirus*, and that it is closely related to HHV-6 and HHV-7 [25]. Since VZV, EBV, and HCMV are not so closely related, an alignment of the sequences of the gB protein used for our Western blot analysis was only possible for HHV-6 and HHV-7 (Figure 3).

### 3.3. Human Antibodies against PCMV and HCMV

Although HCMV is not closely related to PCMV—it has only a similar name—we nevertheless analyzed whether antibodies in human sera specific for PCMV are the result of an infection of humans with this porcine virus, or whether there may be a cross-reactivity with HCMV. For this, human sera reacting positive or negative against PCMV were tested for antibodies against HCMV. No correlation between the reactivity against PCMV and HCMV was found (Table 2), clearly indicating that there is no cross-reactivity between sera against both viruses.

Only in two cases (20%) were identical results found in the testing for both viruses; in 80% no correlation was observed. 

**Figure 4:**
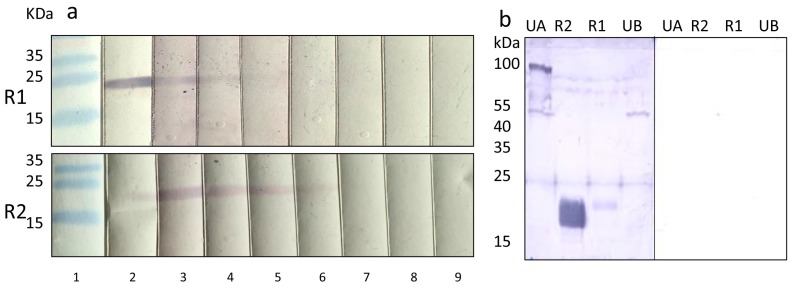
(**a**) Strong reaction of a Cytotect preparation against the recombinant gB proteins R1 and R2 of PCMV. 1, marker, 2, anti-His-tag antibody 1:1000, anti-mouse antibodies 1:1000 as positive controls, 3–9, dilution of the Cytotect preparation starting with 1:50, anti-human antibodies 1:1000; (**b**) left, reaction of the Cytotect preparation (1:50) with the gB proteins R1 and R2, and two tegument proteins of PCMV, UA, and UB; right, negative control, secondary human antibody 1:1000 only.

Most interestingly, when a preparation of Cytotect was tested in the Western blot analysis with the recombinant proteins R1 and R2, a strong reaction was observed (Figure 4a). Cytotect is a commercial anti-HCMV polyclonal immunoglobulin preparation from human plasma from HCMV-positive individuals used for the treatment of HCMV infections in various clinical settings [26], as well as to prevent HCMV transmission to the fetus during pregnancy [27]. Titers of 1:200 in the case of protein R1 as an antigen and 1:400 in the case of protein R2 were observed (Figure 4a). Cytotect also reacted weakly with the two tegument proteins of PCMV (Figure 4b).

However, when 15 sera from humans with a well characterized status of HCMV immunoglobulin were analysed in an Western blot assay using R1 and R2 of PCMV as antigens, no correlation was observed (Table 3). Only in 4 cases (26%) identical results were obtained when testing for both viruses, in 74% no correlation was observed.

### 3.4. Pig Sera Reacting with PCMV Reacted with HHV-6

**Figure 5:**
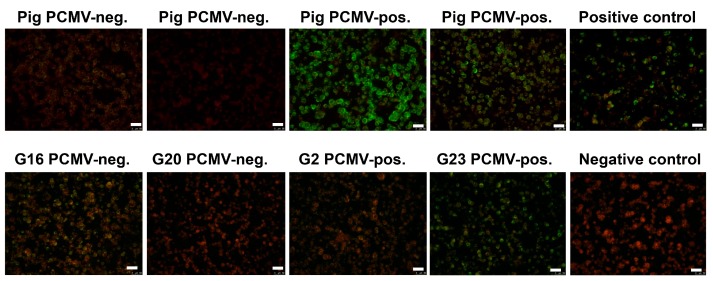
Results of the immunofluorescence analysis of pig and human sera for antibodies reacting against HHV-6. HHV-6 infected cells were used as antigen. In the upper row the reactivity of four pig sera is shown, two of them were PCMV-negative two were PCMV-positive. The “positive control” and the “negative control” are human sera with a known seroreactivity. In the upper row four human sera were analyzed which in parallel were tested on a Western blot analysis using four recombinant proteins of PCMV (Table 5). The bar corresponds to 50 µm.

Since there was no correlation between the reactivity against PCMV and HCMV (HHV-5), confirming that PCMV and HCMV were not closely related and knowing that PCMV is closer related to HHV-6 and HHV-7 [3], sera from pigs reacting against PCMV were screened for a reactivity against HHV-6 using an immunofluorescence assay. Five pig sera positive for PCMV reacted positive in the immunofluorescence assay for HHV-6, five pig sera negative for PCMV reacted negative (Figure 5 shows four of them). These data show that pig sera positively reacting against recombinant PCMV proteins in an Western blot analysis also reacted positively in an immunofluorescence assay against HHV-6, indicating a cross-reactivity and 100% correlation. 

### 3.5. Human Sera Reacting with HHV-6 Reacted with PCMVand Vice Versa

Furthermore, when sera from humans reacting positively or negatively against HHV-6 were tested for reactivity against PCMV, a good correlation was observed (Table 4). Two sera samples negative for HHV-6 were negative for PCMV, and out of four sera samples positive for HHV-6, only one did not react against PCMV, a serum with a low titer (1:32) against HHV-6. The human serum 6331 with the highest titer against HHV-6 reacted strongly against PCMV (Figure 1b, Table 4). On the other hand, all human sera reacting positively in a Western blot analysis with recombinant PCMV proteins also reacted positively in an immunofluorescence assay for HHV-6, with one exception—serum G2 (Table 5). The fact that all sera samples that were negative for PCMV also reacted with HHV-6 is not surprising, given the high prevalence of HHV-6 in the human population.

## 4. Discussion

Porcine cytomegalovirus is one of the pathogens that should be eliminated from pigs intended for use as organ donors in xenotransplantation. For this purpose, reliable diagnostic test systems are needed, and recently sensitive PCR-based [28,29,30,31,32] as well as serological assays [14,33] have been developed.

The positive Western blot result when testing human sera with recombinant PCMV proteins was an interesting finding, which allowed to us speculate that PCMV may be able to infect humans. One porcine virus that frequently infects humans is the hepatitis E virus (HEV), genotype 3 (gt3) (for review see [34]). In most cases, this infection is asymptomatic and in certain regions in France up to 56% of the population have antibodies against HEV, indicating a previous infection due to the consumption of undercooked liver sausages called figatellu [35,36]. HEV is also transmitted to humans by contact with pigs [34], and from human to human by organ transplants or blood donations [34]. Severe hepatitis was observed mostly in patients with underlying liver disease and in immunosuppressed individuals where HEV can cause a chronic or even fatal disease [34]. 

When analyzing the reason for the presence of reactive antibodies against PCMV in human sera, a cross-reactivity with antibodies against HCMV and other herpesviruses, with the exception of HHV-6 and HHV-7, can be excluded due to a lack of sequence homology [29]. In the case of HCMV, this was confirmed experimentally by testing sera positive for antibodies against PCMV and HCMV in different assays (Table 2 and Table 3). In contrast, a cross-reactivity with HHV-6 was shown, since pig sera reacting against PCMV also reacted with human cells infected with HHV-6, and human sera reacting with HHV-6 recognized recombinant PCMV proteins (Figure 5, Table 4 and Table 5). The fact that Cytotect, an immunoglobulin preparation from HCMV-positive individuals, reacted with the PCMV proteins can certainly be explained by the presence of antibodies against HHV-6 in the preparation. HHV-6A and HHV-6B infect nearly the entire human population that has been tested. While HHV-6B is present in almost 100% of the world’s population, HHV-6A appears to be less frequent in Japan, North America, and Europe [37]. 

It is well known that HHV-6 and HHV-7 are close relatives of PCMV [3]. In a comparison of PCMV gB with the corresponding region of other herpesviruses, the highest identities were found with human herpesviruses 6 and 7 (HHV-6 and -7; 43.4% and 42.6%, respectively) [25]. Also in phylogenetic analysis, the PCMV gB clustered with HHV-6 and HHV-7. Despite a considerable intra-species variation (between the complete gB sequences of five different PCMV strains and isolates from the United Kingdom, Germany, Spain, Japan, and Sweden, differences of 3.4% were found), similar sequences exist in the gB of PCMV and HHV-6 (Figure 3), which could allow the binding of cross-reacting antibodies. Regions of up to seven identical or conserved amino acids were detected (Figure 4), which exceeds the size of common epitopes of approximately five amino acids [38]. Since PCMV is also closely related to HHV-7, and a high homology in the sequence of the antigens used in Western blot analyses was found (Figure 3), we cannot exclude a cross-reactivity with HHV-7. Unfortunately, reliable detection methods for HHV-7 are not available and at hand, and in an immunofluorescence assay on the basis of HHV-7-infected cells, no specific reaction could be observed due to a high unspecific background.

HHV-6B is known to be associated with the childhood disease *roseola infantum*, and a spectrum of other clinical diseases ranging from asymptomatic infection to acute febrile illnesses with severe neurological complications [39]. Furthermore, HHV-6A and HHV-6B are associated with diverse complications in transplant patients [40]. Most interestingly, both viruses can integrate their viral sequences into the host cell genome [39]. Although it is unlikely that PCMV can infect human cells and replicate in humans, it is unclear whether PCMV can induce similar diseases as HHV-6 and/or reduce the survival time of the pig transplant. Nevertheless, elimination programs have been developed to eliminate PCMV from pig breeds generated for xenotransplantation. They are based first of all on early weaning, but may include Cesarean delivery or embryo transfer [4,41]. Unfortunately, there are still no effective vaccines to prevent infection [42] or treatments to eliminate PCMV [43]. Antiviral drug efficiently inhibiting HCMV in humans were ineffective against PCMV [44]. 

Herpesviruses are considered to specifically infect their own species [1,8]. However, the question of whether PCMV can infect human cells and replicate in humans is still unanswered. There is one publication reporting the infection of human fibroblasts [9], while co-cultivation of PCMV-infected pig macrophages with two human cell lines (293 and Raji) did not facilitate virus transmission [10]. Our own experiments also showed that human cells cannot be infected by PCMV [45]. Five human cell lines (HeLa, 293T, TZM-bl, Jurkat, and HepG2) were spinoculated and incubated with serum from one PCMV-infected animal. No PCMV was detected in the treated cells or in the pelleted supernatant using a highly sensitive PCR [42]. 

This is the first report demonstrating an antibody cross-reactivity between PCMV and HHV-6. However, this cross-reactivity is not unexpected, considering the sequence homology (Figure 3). In this context, it may be interesting to discuss the question of whether the presence of antibodies against HHV-6 may prevent infection with PCMV.

## Figures and Tables

**Table 1:** Testing of pig and human sera for PCMV by Western blot analysis, in parallel PCMV infection was determined by PCR. n.t., not tested.

	Number Tested	PCR	Western Blot Analysis Using PCMV Proteins			
			R1	R2	UA	UB
Human sera						
Butcher and workers in the meat production industry	24	n.t.	1	3	n.t.	n.t.
Blood donors	24	n.t.	1	0	n.t.	n.t.
Pig sera						
Aachen minipigs	12	2	3	12	6	6
Göttingen minipigs	11	5	2	4	4	9
Slaughterhouse pigs	12	2	4	10	12	9

**Table 2:** Testing human sera for porcine cytomegalovirus (PCMV) and human cytomegalovirus (HCMV) by Western blot analysis.

Serum Number	Western Blot Analysis PCMV ^(1)^		Result HCMV ^(2)^
	R1	R2	
1 ^(**3)**^	−	+/−	>250
2 ^(**3)**^	−	+	>250
3	−	+	-
4	−	+/−	-
5	−	+	-
6	−	−	>250
7	−	−	6.1
8	−	−	>250
9	−	−	214.1
10	−	−	180.7

^(1)^ Serum dilution 1:5/1:10, two recombinant parts of the glycoprotein gB of PCMV, R1 and R2, were used as antigens; ^(2)^ Performed at Labor Berlin, chemiluminescent microparticle immunoassay (CMIA), units; ^(3)^ Gray: Identical results.

**Table 3:** Testing human sera for PCMV and HCMV by Western blot analysis.

Number	Serum Number	HCMV IgG ^(1)^	Western Blot Analysis Using PCMV gB Antigens ^(2)^	
			R1	R2
1	914,640	−	−	+
2 ^(3)^	914,644	−	−	−
3 ^(3)^	914,672	+	−	+
4	914,714	−	−	+
5	914,814	+	−	−
6	914,854	−		+
7	914,855	+	−	−
8	914,887	−	−	+
9	914,978	+	−	−
10	914,984	+	−	−
11 ^(3)^	914,986	+	−	+
12	914,998	+	−	−
13	915,159	+	−	−
14 ^(3)^	915,164	+	−	+
15	915,274	+	−	−

^(1)^ Using the LIAISON CMV IgG II assay; ^(2)^ All sera were diluted 1:150; ^(3)^ Gray: identical result in PCMV (R2) and HCMV testing.

**Table 4:** Correlation between the reactivity against porcine cytomegalovirus (PCMV) and human herpesvirus-6 (HHV-6).

Number	Serum Number	HHV-6 ^(1)^	PCMV Western Blot Analysis			
			R1	R2	U54A	U54B
1	11,415	−	−	−	−	−
2	10,301	−	−	−	−	−
3	9858	32	−	−	−	−
4	7186	>64	−	+	−	−
5	6515	32	−	+	−	−
6	6331	>64	−	+	+	+

^(1)^ Immunofluorescence testing.

**Table 5:** Testing human sera for PCMV and HHV-6 by Western blot analysis and immunofluorescence assay.

Number	Serum Number	HHV-6 ^(1)^	PCMV Western Blot Analysis			
			R1	R2	U54A	U54B
PCMV-positive sera						
1	G2	−	−	+	−	+
2	G13	>64	−	+	−	+
3	G15	16	−	+	+	−
4	G23	>>64	+/−	+	+	+
5	G24	16	−	+	−	−
PCMV-negative sera						
1	G16	>64	−	−	−	−
2	G17	>64	−	−	−	−
3	G18	>64	−	−	−	−
4	G19	>>64	−	−	−	−
5	G20	−	−	−	−	+

^(1)^ Immunofluorescence testing.

## References

[1] Davison A.J. (2010). Herpesvirus systematics. Vet. Microbiol..

[2] Mettenleiter T.C., Keil G.M., Fuchs W., Mettenleiter T.C., Sobrino F. (2008). Molecular Biology of Animal Herpesviruses. Animal Viruses: Molecular Biology.

[3] Gu W., Zeng N., Zhou L., Ge X., Guo X., Yang H. (2014). Genomic organization and molecular characterization of porcine cytomegalovirus. Virology.

[4] Denner J. (2015). Xenotransplantation and porcine cytomegalovirus. Xenotransplantation.

[5] Puga Yung G.L., Rieben R., Bühler L., Schuurman H.J., Seebach J. (2017). Xenotransplantation: Where do we stand in 2016. Swiss Med. Wkly..

[6] Denner J. (2016). Recent Progress in Xenotransplantation, with Emphasis on Virological Safety. Ann. Transplant..

[7] Ramanan P., Razonable R.R. (2013). Cytomegalovirus infections in solid organ transplantation: A review. Infect. Chemother..

[8] Jurak I., Brune W. (2006). Induction of apoptosis limits cytomegalovirus cross-species infection. EMBO J..

[9] Whitteker J.L., Dudani A.K., Tackaberry E.S. (2008). Human fibroblasts are permissive for porcine cytomegalovirus in vitro. Transplantation.

[10] Tucker A.W., Galbraith D., McEwan P., Onions D. (1999). Evaluation of porcine cytomegalovirus as a potential zoonotic agent in Xenotransplantation. Transplant. Proc..

[11] Yamada K., Tasaki M., Sekijima M., Wilkinson R.A., Villani V., Moran S.G., Cormack T.A., Hanekamp I.M., Hawley R.J., Arn J.S. (2014). Porcine cytomegalovirus infection is associated with early rejection of kidney grafts in a pig to baboon xenotransplantation model. Transplantation.

[12] Sekijima M., Waki S., Sahara H., Tasaki M., Wilkinson R.A., Villani V., Shimatsu Y., Nakano K., Matsunari H., Nagashima H. (2014). Results of life-supporting galactosyltransferase knockout kidneys in cynomolgus monkeys using two different sources of galactosyltransferase knockout swine. Transplantation.

[13] Mueller N.J., Kuwaki K., Dor F.J., Knosalla C., Gollackner B., Wilkinson R.A., Sachs D.H., Cooper D.K., Fishman J.A. (2004). Reduction of consumptive coagulopathy using porcine cytomegalovirus-free cardiac porcine grafts in pig-to-primate xenotransplantation. Transplantation.

[14] Plotzki E., Keller M., Ivanusic D., Denner J. (2016). A new Western blot assay for the detection of porcine cytomegalovirus (PCMV). J. Immunol. Methods.

[15] Grote A., Hiller K., Scheer M., Munch R., Nortemann B., Hempel D.C., Jahn D. (2005). JCat: A novel tool to adapt codon usage of a target gene to its potential expression host. Nucleic Acids Res..

[16] Plotzki E., Keller M., Ehlers B., Denner J. (2016). Immunological methods for the detection of porcine lymphotropic herpesviruses (PLHV). J. Virol. Methods.

[17] Plotzki E., Heinrichs G., Kubícková B., Ulrich R.G., Denner J. (2016). Microbiological characterization of a newly established pig breed, Aachen Minipigs. Xenotransplantation.

[18] Tacke S.J., Bodusch K., Berg A., Denner J. (2001). Sensitive and specific immunological detection methods for porcine endogenous retroviruses applicable to experimental and clinical xenotransplantation. Xenotransplantation.

[19] DNASTAR. www.dnastar.com.

[20] Neipel F., Ellinger K., Fleckenstein B. (1992). Gene for the major antigenic structural protein (p100) of human herpesvirus-6. J. Virol..

[21] Mueller N.J., Livingston C., Knosalla C., Barth R.N., Yamamoto S., Gollackner B., Dor F.J., Buhler L., Sachs D.H., Yamada K. (2004). Activation of porcine cytomegalovirus, but not porcine lymphotropic herpesvirus, in a pig-to-baboon xenotransplantation. J. Infect. Dis..

[22] Duvigneau J.C., Hartl R.T., Groiss S., Gemeiner M. (2005). Quantitative simultaneous multiplex real-time PCR for the detection of porcine cytokines. J. Virol. Methods.

[23] Arvin A., Campadelli-Fiume G., Mocarski E., Moore P.S., Roizman B., Whitley R., Yamanishi K. (2007). Human Herpesviruses: Biology, Therapy, and Immunoprophylaxis.

[24] Miller C.S., Berger J.R., Mootoor Y., Avdiushko S.A., Zhu H., Kryscio R.J. (2006). High prevalence of multiple human herpesviruses in saliva from human immunodeficiency virus-infected persons in the era of highly active antiretroviral therapy. J. Clin. Microbiol..

[25] Widen F., Goltz M., Wittenbrink N., Ehlers B., Banks M., Belak S. (2001). Identification and sequence analysis of the glycoprotein B gene of porcine cytomegalovirus. Virus Genes.

[26] Rea F., Potena L., Yonan N., Wagner F., Calabrese F. (2016). Cytomegalovirus Hyper Immunoglobulin for CMV Prophylaxis in Thoracic Transplantation. Transplantation.

[27] Buxmann H., Stackelberg O.M., Schlößer R.L., Enders G., Gonser M., Meyer-Wittkopf M., Hamprecht K., Enders M. (2012). Use of cytomegalovirus hyperimmunoglobulin for prevention of congenital cytomegalovirus disease: A retrospective analysis. J. Perinat. Med..

[28] Hamel A.L., Lin L., Sachvie C., Grudeski E., Nayar G.P. (1999). PCR assay for detecting porcine cytomegalovirus. J. Clin. Microbiol..

[29] Widen B.F., Lowings J.P., Belak S., Banks M. (1999). Development of a PCR system for porcine cytomegalovirus detection and determination of the putative partial sequence of its DNA polymerase gene. Epidemiol. Infect..

[30] Fryer D., Fishman J.A., Emery V.C., Clark D.A. (2001). Quantitation of porcine cytomegalovirus in pig tissues by PCR. J. Clin. Microbiol..

[31] Lee C.S., Moon H.J., Yang J.S., Park S.J., Song D.S., Kang B.K., Park B.K. (2007). Multiplex PCR for the simultaneous detection of pseudorabies virus, porcine cytomegalovirus, and porcine circovirus in pigs. J. Virol. Methods.

[32] Morozov V.A., Morozov A.V., Denner J. (2016). New PCR diagnostic systems for the detection and quantification of porcine cytomegalovirus (PCMV). Arch. Virol..

[33] Liu X., Zhu L., Shi X., Xu Z., Mei M., Xu W., Zhou Y., Guo W., Wang X. (2012). Indirect-blocking ELISA for detecting antibodies against glycoprotein B (gB) of porcine cytomegalovirus (PCMV). J. Virol. Methods.

[34] Denner J. (2015). Xenotransplantation and Hepatitis E virus. Xenotransplantation.

[35] Colson P., Borentain P., Queyriaux B., Kaba M., Moal V., Gallian P., Heyries L., Raoult D., Gerolami R. (2010). Pig liver sausage as a source of hepatitis E virus transmission to humans. J. Infect. Dis..

[36] Renou C., Roque Afonso A.M., Pavio N. (2014). Foodborne Transmission of Hepatitis E Virus from Raw Pork Liver Sausage, France. Emerg. Infect. Dis..

[37] Braun D.K., Dominguez G., Pellett P.E. (1997). Human herpesvirus 6. Clin. Microbiol. Rev..

[38] Kaulitz D., Fiebig U., Eschricht M., Wurzbacher C., Kurth R., Denner J. (2011). Generation of neutralising antibodies against porcine endogenous retroviruses (PERVs). Virology.

[39] Tesini B.L., Epstein L.G., Caserta M.T. (2014). Clinical impact of primary infection with roseoloviruses. Curr. Opin. Virol..

[40] Hill J.A., Zerr D.M. (2014). Roseoloviruses in transplant recipients: Clinical consequences and prospects for treatment and prevention trials. Curr. Opin. Virol..

[41] Tucker A.W., McNeilly F., Meehan B., Galbraith D., McArdle P.D., Allan G., Patience C. (2003). Methods for the exclusion of circoviruses and gammaherpesviruses from pigs. Xenotransplantation.

[42] Schleiss M.R. (2013). Developing a Vaccine against Congenital Cytomegalovirus (CMV) Infection: What Have We Learned from Animal Models? Where Should We Go Next?. Future Virol..

[43] Fryer J.F., Griffiths P.D., Emery V.C., Clark D.A. (2004). Susceptibility of porcine cytomegalovirus to antiviral drugs. J. Antimicrob. Chemother..

[44] Mueller N.J., Sulling K., Gollackner B., Yamamoto S., Knosalla C., Wilkinson R.A., Kaur A., Sachs D.H., Yamada K., Cooper D.K. (2003). Reduced efficacy of ganciclovir against porcine and baboon cytomegalovirus in pig-to-baboon xenotransplantation. Am. J. Transplant..

[45] Morozov V.A., Denner J. (2017). Personal communication.

